# The role of beta-lactamase-producing-bacteria in mixed infections

**DOI:** 10.1186/1471-2334-9-202

**Published:** 2009-12-14

**Authors:** Itzhak Brook

**Affiliations:** 1 Department of Pediatrics, Georgetown University School of Medicine, Washington, DC, USA

## Abstract

Beta-lactamase-producing bacteria (BLPB) can play an important role in polymicrobial infections. They can have a direct pathogenic impact in causing the infection as well as an indirect effect through their ability to produce the enzyme beta-lactamase. BLPB may not only survive penicillin therapy but can also, as was demonstrated in *in vitro *and *in vivo *studies, protect other penicillin-susceptible bacteria from penicillin by releasing the free enzyme into their environment. This phenomenon occurs in upper respiratory tract, skin, soft tissue, surgical and other infections. The clinical, *in vitro*, and *in vivo *evidence supporting the role of these organisms in the increased failure rate of penicillin in eradication of these infections and the implication of that increased rate on the management of infections is discussed.

## Review

Penicillins have been the agents of choice for the therapy of a variety of bacterial infections. However, within the past sixty years, an increased resistance to these drugs has been noted. In addition to bacteria long known to resistant penicillin, such as *Staphylococcus aureus *and *Enterobacteriaceae*, other previously susceptible organisms became increasingly resistant due to several mechanisms including the production of the enzyme beta-lactamase (BL). These include aerobic and facultative bacteria such as *Haemophilus influenzae, Moraxella catarrhalis*, as well as anaerobic Gram-negative bacilli (AGNB, i.e. *Bacteroides fragilis *group, pigmented *Prevotella *and *Porphyromonas, Prevotella bivia*, and *Prevotella disiens*) and *Fusobacterium *spp.) [1-3].

**Figure 1:**
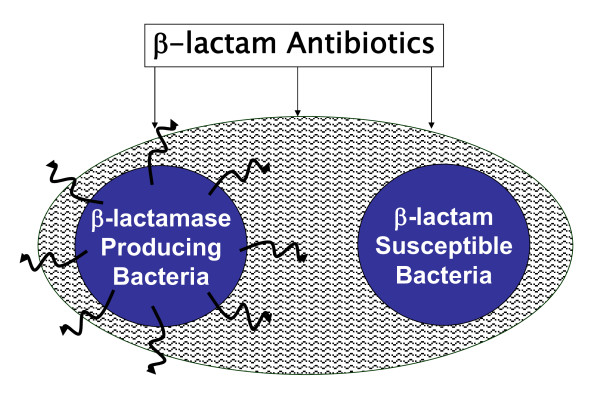
**Protection of penicillin-susceptible bacteria from penicillin by beta-lactamase -producing bacteria**.

Beta-lactamase-producing bacteria (BLPB) may have an important clinical role in infections. These organisms can be pathogenic in causing the infection as well as have an indirect effect through their ability to produce the enzyme BL into their environment. BLPB may not only survive penicillin therapy but also may protect other penicillin-susceptible bacteria from penicillins by releasing the free enzyme into their environment (Figure 1)[4].

*In vivo *and *in vitro *studies have demonstrated this phenomenon. Animal studies demonstrated the ability of the enzyme BL to influence polymicrobial infections. BL producing of AGNB protected a penicillin-sensitive *Fusobacterium necrophorum *[5] and Group A beta hemolytic streptococci (GABHS) [6] from penicillin therapy in mice. Clindamycin or the combination of penicillin and clavulanate (a BL inhibitor), which are active against both GABHS and AGNB, were effective in eradicating the infection [7]. An increase in resistance of GABHS to penicillin was found when it was co-inoculated with *S. aureus *[8], *Haemophilus parainfluenzae *[9], or *B. fragilis *[10].

Several studies demonstrate the activity of the enzyme BL in polymicrobial infections. Penicillins were degraded by purulent exudates obtained from abscesses [11,12] and in experimental *B. fragilis *infection [13].

The presence of BL in clinical specimens was reported in abscesses and mixed infections. These include abdominal infections [12], empyema [14], cerebrospinal specimens [15], abscesses [16], ear aspirates of acute and chronic ear infections [17,18], and aspirates of acutely and chronically inflamed maxillary sinuses. Many of these infections had failed beta lactam therapies and required surgical drainage to enhance cure [19].

The isolation of penicillin-susceptible bacteria mixed with BLPB in patients who have failed to respond to penicillin or cephalosporin therapy suggests the ability of BLPB to protect a penicillin-susceptible or cephalosporin-susceptible organism from the activity of those drugs.

The emergence of oral BLPB was shown to be associated with the administration of penicillin therapy [20]. The selection of BLPB following antimicrobial therapy may account for many of the clinical failures that occur after penicillin therapy [21]. BLPB were recovered in 75 (40%) of 185 children with orofacial and respiratory infections who failed to respond to penicillin [22].

Aerobic and anaerobic BLPB may play a role in penicillin failure to eradicate GABHS tonsillitis [8,9,21-31]. It is plausible that these BLPB can protect GABHS from penicillin by inactivation of the antibiotic. (Figure 1) BLPB were recovered in 37 of 50 tonsils (74%) removed from children who failed penicillin therapy. These observations were confirmed by Reilly et al. [29], Chagollan et al. [30], and Tuner and Nord [31]. Assays of the free enzyme in the tissues demonstrated its presence in 33 of 39 (85%) tonsils that harbored BLPB [28].

BLPB emerged in the oropharynx promptly following penicillin therapy [32-34]. BLPB were isolated in 3 of 21 (14%) of children prior to penicillin therapy, and in 10 of 21 (48%) following one course of penicillin [33]. In a study of 26 children who were treated with penicillin for seven days 11% harbored BLPB prior to the thearpy which increased to 45% at the conclusion of the treatment, and the incidence was 27% three months later [34]. These organisms were also isolated from household contacts of children repeatedly treated with penicillin, suggesting their possible transfer within a family [33].

Chemoprophylaxis of 20 children with recurrent otitis media with amoxicillin increased the recovery rate of BLPB from 20% to 100% after six month [35]. No change occurred in the recovery of BLPB in a group of 20 children who received sulfisoxazole.

An association has been noted between the presence of BLPB even prior to therapy of acute GABHS tonsillitis and the outcome of 10-day oral penicillin therapy [36]. Of 98 children with acute GABHS tonsillitis, 36 failed to respond to therapy. Prior to therapy, 18 isolates of BLPB were detected in 16 (26%) of those cured and following therapy 30 such organisms were recovered in 19 (31%) of these children. In contrast, prior to therapy, 40 BLPB were recovered from 25 (69%) of the children who failed, and following therapy, 62 such organisms were found in 31 (86%) of the children in that group.

A high levels of BL in saliva reflects colonization with many BLPB [37]. Previous antimicrobial therapy can select for resistant bacterial strains that could persist in the nasopharynx to re-emerge in new ear and sinus infection [38].

The presence of BLPB in mixed infection warrants administration of drugs that will be effective in eradication of BLPB as well as the other pathogens. The high failure rate of penicillin therapy associated with the recovery of BLPB in a growing number of cases of mixed aerobic-anaerobic infections highlights the importance of this therapeutic approach [21,22].

An infection in which this therapeutic approach has been successful is recurrent tonsillitis [39-51]. Antimicrobials active against aerobic and anaerobic BLPB as well as GABHS were more effective in the eradication of this infection and even prevented elective tonsillectomy [47] compared to penicillin. These include lincomycin [39-42], clindamycin [43-48], and amoxicillin/clavulanate [52].

BLPB colonized over 83% of the adenoids in children with chronic adeno-tonsillitis [53] which may explain the persistence of many pathogens including *Streptococcus pneumoniae*. The total number of potential pathogens and BLPB were lower in those treated with amoxicillin/clavulante or clindamycin [54,55] Similarly amoxicillin/clavulante was superior to amoxicillin in achieving clinical cure (92% vs 64%) and reducing the number of potential nasopharyngeal pathogens including *S. pneumoniae *and BLPB in children with acute otitis media [56].

Two studies illustrated the superiority of clindamycin to penicillin in the treatment of lung abscesses [57,58]. This was postulated to be due to its ability to eradicate the anaerobic BLPB present in lung abscess.

Antimicrobials effective against anaerobic BLPB (ticarcillin/clavulanate or clindamycin with ceftazidime) were superior to an agent without such coverage (ceftriaxone) in the therapy of aspiration or tracheostomy-associated pneumonia in 57 children [59].

## Conclusions

The above studies illustrate that the successful management of polymicrobial infections is enhanced by directing antimicrobial therapy at the eradication of both aerobic and anaerobic BLPB. This approach is also useful in management infections such as tonsillitis where BLPB are part of the normal flora at the infection site and is often employed it the treatment of other infections at all body sites. Some of these are polymicrobial where one of the pathogens is a BLPB while in others the role of the BLPB as a primary pathogen is unclear (i. e. tonsillitis).

Although beta lactam antibiotics are still the mainstay in treatment of numerous infections, agents effective against BLPB should be considered in the treatment of those who failed these agents. Since BLPB can spread within the community as well as the hospital efforts should be made to reduce the spread.^33 ^However, further studies are warranted to critically investigate these modalities.

## Abbreviations

BL: Beta lactamase; BLPB: Beta lactamase producing bacteria; GABHS: group A Beta hemolytic bacteria.

## Competing interests

The authors declare that they have no competing interests.

## Pre-publication history

The pre-publication history for this paper can be accessed here:

http://www.biomedcentral.com/1471-2334/9/202/prepub
